# Coronavirus endoribonuclease targets viral polyuridine sequences to evade activating host sensors

**DOI:** 10.1073/pnas.1921485117

**Published:** 2020-03-20

**Authors:** Matthew Hackbart, Xufang Deng, Susan C. Baker

**Affiliations:** ^a^Department of Microbiology and Immunology, Stritch School of Medicine, Loyola University Chicago, Maywood, IL 60153

**Keywords:** coronavirus, endoribonuclease, EndoU, nsp15, interferon

## Abstract

Coronaviruses (CoVs) are positive-sense RNA viruses that can emerge from endemic reservoirs and infect zoonotically, causing significant morbidity and mortality. CoVs encode an endoribonuclease designated EndoU that facilitates evasion of host pattern recognition receptor MDA5, but the target of EndoU activity was not known. Here, we report that EndoU cleaves the 5′-polyuridines from negative-sense viral RNA, termed PUN RNA, which is the product of polyA-templated RNA synthesis. Using a virus containing an EndoU catalytic-inactive mutation, we detected a higher abundance of PUN RNA in the cytoplasm compared to wild-type−infected cells. Furthermore, we found that transfecting PUN RNA into cells stimulates a robust, MDA5-dependent interferon response, and that removal of the polyuridine extension on the RNA dampens the response. Overall, the results of this study reveal the PUN RNA to be a CoV MDA5-dependent pathogen-associated molecular pattern (PAMP). We also establish a mechanism for EndoU activity to cleave and limit the accumulation of this PAMP. Since EndoU activity is highly conserved in all CoVs, inhibiting this activity may serve as an approach for therapeutic interventions against existing and emerging CoV infections.

## Significance

Cells carry sensors that are primed to detect invading viruses. To avoid being recognized, coronaviruses express factors that interfere with host immune sensing pathways. Previous studies revealed that a coronavirus endoribonuclease (EndoU) delays activation of the host sensor system, but the mechanism was not known. Here, we report that EndoU cleaves a viral polyuridine sequence that would otherwise activate host immune sensors. This information may be used in developing inhibitors that target EndoU activity and prevent diseases caused by coronaviruses.

Coronaviruses (CoVs) are positive-sense RNA viruses that replicate in the cytoplasm of infected cells. The positive-sense virion RNA is translated to generate the viral replication machinery, which then replicates the positive-sense RNA into negative-sense, genomic RNA and subgenomic RNAs (sgRNAs). The negative-sense RNAs then function as templates for synthesis of positive-sense genomic RNA and sgRNA (1, 2). This replication strategy can generate long double-stranded RNA (dsRNA) intermediates (3), that may act as pathogen-associated molecular patterns (PAMPs) recognized by cytoplasmic pattern recognition receptors (PRRs) (4, 5). The specific PRR that recognizes CoV RNA is MDA5, which can activate the type I interferon (IFN) response in macrophages (6). Other host dsRNA PRRs, such as PKR and OAS, are also activated and operate to limit CoV replication (7–11). CoVs encode multiple proteins that antagonize these innate immune responses, particularly the activation of the IFN response (9, 12–16), ultimately leading to a dysregulated immune response and increased immunopathogenesis (17, 18). Understanding the mechanisms used by CoVs to delay IFN signaling may provide opportunities for the development of antivirals and live-attenuated vaccines to limit CoV infections.

Here, we investigate the mechanism used by one CoV IFN antagonist, the nonstructural protein 15 (nsp15), which is an endoribonuclease designated EndoU. EndoU is highly conserved in all known CoVs (19, 20). EndoU is similar to the cellular endoribonuclease XendoU, as revealed by bioinformatic analysis of the amino acid sequence (21). X-ray structures of EndoU revealed conserved endoribonuclease folds with catalytic histidine residues required for endoribonuclease activity (22–26). Purified EndoU was shown to cleave single-stranded RNA and dsRNA at uridine residues in in vitro assays (22, 25, 27–30). However, the target of EndoU activity during viral infection was unknown. Initial studies revealed that EndoU colocalizes with the viral replication complex (31, 32), and it was suggested that EndoU was necessary for efficient virus RNA replication in cell culture (28, 29). More recent findings, however, revealed that EndoU catalytic mutant (EndoUmut) viruses replicate as well as wild-type virus in IFN-nonresponsive cells, but are severely impaired for replication in IFN-responsive macrophages (10, 11). These recent results revealed that EndoU activity is important for limiting the sensing of viral RNA by host dsRNA sensors such as MDA5, PKR, and OAS/RNaseL. Limiting viral RNA recognition contributes to delayed type I IFN responses; thus viruses with intact EndoU activity are more virulent than their EndoU-mutant counterparts (10, 11, 20).

In this study, we show that CoV EndoU activity limits the abundance and length of the polyuridine (polyU) extension on 5′-polyU-containing, negative-sense (PUN) RNAs for both the beta-CoV mouse hepatitis virus strain A59 (MHV-A59) and the alpha-CoV PEDV. Importantly, we found that the PUN RNAs can act as PAMPs recognized by MDA5. Overall, we propose a mechanism for EndoU, which is to cleave polyU sequences from PUN RNAs, thus limiting the formation of a PAMP and impeding the ability of MDA5 to activate the innate immune response to infection.

## Results

### EndoU Activity Reduces the Accumulation of an Epitope Recognized by an Anti-dsRNA Antibody in CoV-Infected Hepatocytes.

**Fig. 1.**
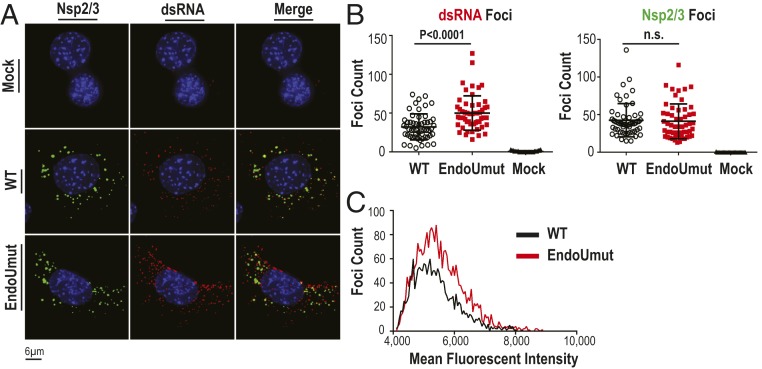
Evaluating the accumulation of an epitope recognized by K1 antibody in virus-infected AML12 hepatocytes. AML12 hepatocytes were infected with wild-type (WT) or EndoUmut MHV at an MOI of 0.1. Cells were fixed at 8 hpi and stained with K1 anti-dsRNA antibody, anti-nsp2/3, and Hoescht 33342 nuclei stain. (*A*) Subcellular localization of dsRNA and nsp2/3 foci was visualized. (*B*) Foci for (*Left*) dsRNA and (*Right*) nsp2/3 were quantified using Imaris software from 50 individual cells. (*C*) The median fluorescent intensity was calculated for each individual dsRNA foci and compared between WT and EndoUmut infections. Values were analyzed by Student *t* tests. Data are representative of three independent experiments and presented as individual cell points with mean ± SD; n.s., not significant.

Previously, we reported that EndoU activity delays the accumulation of an epitope recognized by the K1 antibody in the cytoplasm of IFNAR^−/−^ bone marrow-derived macrophages (BMDMs) as measured by immunofluorescence (11). The K1 antibody was shown to recognize dsRNA; therefore, we hypothesized that the CoV epitope was dsRNA. To determine whether this phenotype is present in a stable cell line, we infected IFN-responsive AML12 hepatocytes with wild-type or EndoUmut MHV and measured accumulation of replication complexes (anti-nsp2/3) and dsRNA foci (anti-dsRNA, K1) at 8 h postinfection (hpi) (Fig. 1*A*). In AML12 cells, wild-type and EndoUmut virus have similar replication kinetics and viral RNA expression, but EndoUmut elicited enhanced type I and type III IFN expression during infection (*SI Appendix*, Fig. S1). We quantified the number of nsp2/3 foci and dsRNA foci from 50 individual cells. We found that, while the numbers of nsp2/3-labeled replication complexes were not significantly different (Fig. 1*B*, *Right*), the total number of dsRNA foci per cell was elevated in EndoUmut-infected cells (Fig. 1*B*, *Left*). Median fluorescent intensity of the individual dsRNA foci was also brighter in EndoUmut-infected cells (Fig. 1*C*). These results indicate that EndoUmut infection results in increased abundance of an epitope recognized by the K1 anti-dsRNA antibody.

### The Viral RNA Recognized by the K1 Antibody during CoV Infection Is Negative-Sense RNA.

**Fig. 2.**
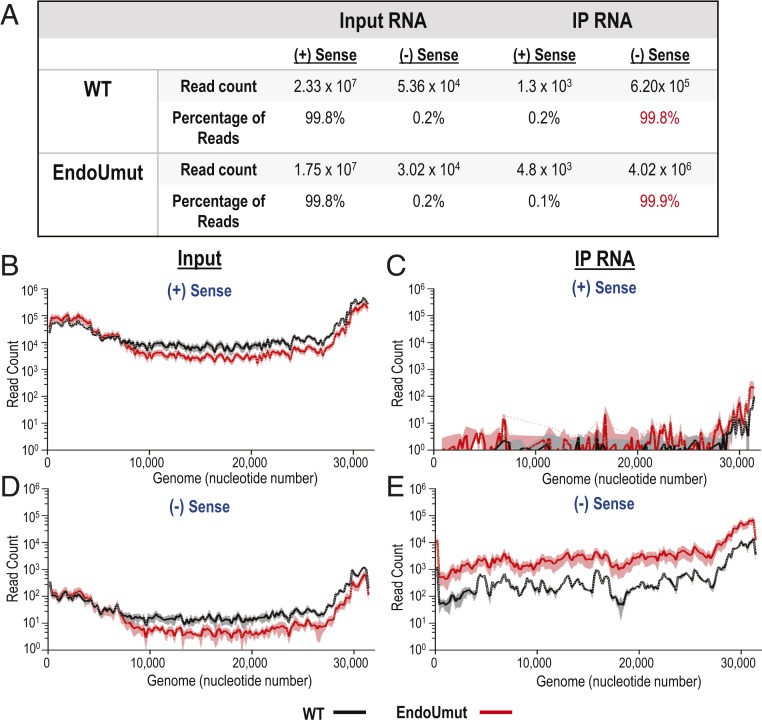
RNA-seq analysis of input viral RNA and RNA immunoprecipitated with anti-dsRNA antibody K1. IFNAR^−/−^ BMDMs were infected with WT or EndoUmut virus at an MOI of 1. At 6 hpi, RNA was purified, mixed with anti-dsRNA antibody, precipitated with protein G beads, and purified off the beads. Input RNA and immunoprecipitated RNA samples were evaluated by RNA-seq. (*A*) Summary of RNA reads mapped to MHV-A59 genome. Values in tables are the means of three biological triplicates. (*B*–*E*) Total reads mapped to the viral genome. Reads were mapped to the positive-sense (+) RNA from (*B*) input RNA and (*C*) immunoprecipitated RNA. Reads were mapped to the negative-sense (−) RNA from (*D*) input RNA and (*E*) immunoprecipitated RNA. Read counts were quantified for each nucleotide of the genome, then averaged into bins of 200 nucleotides for three biological triplicates. The black (WT) and red (EndoUmut) lines represent the mean of each bin, and shaded regions are the SD from the mean. Data are representative of two independent experiments.

Since the RNA bound by the K1 antibody accumulates in the absence of EndoU activity, we sought to identify this RNA. To this end, we sequenced the RNA precipitated with the K1 anti-dsRNA antibody. We obtained ∼30 million reads for each total RNA sample and ∼10 million reads for immunoprecipitated samples. Upon mapping the reads to the mouse genome, we found similar read counts to host genes from both wild-type− and EndoUmut-infected cells (data available at NCBI GEO database, accession no. GSE144886) (33). We then mapped the reads to the MHV-A59 genome (GenBank accession no. AY910861) (34), and separated the viral reads by strand specificity, expecting to identify complementary sequences from positive- and negative-sense RNA. Surprisingly, we found that the majority of reads from the immunoprecipitated RNA sample mapped to negative-sense RNA (Fig. 2*A*). We discovered that 99.8% of the reads from the input RNA sample mapped to positive-sense RNA. In contrast, 99.8% of the reads from the immunoprecipitated RNA mapped to negative-sense RNA. We found that the reads from the input RNA sample mapped across the entire MHV genome, as expected (Fig. 2 *B* and *D*). Similarly, the reads from the immunoprecipitated RNA sample also mapped across the entire genome (Fig. 2 *C* and *E*). We concluded that the K1 antibody immunoprecipitated full-length, negative-sense RNAs. When comparing the read counts between wild-type virus- and EndoUmut virus-infected samples, we found an eightfold increase (6 × 10^5^ read counts versus 4 × 10^6^ read counts) in the abundance of the reads from the EndoUmut virus-infected samples (Fig. 2*A*). These results are consistent with the increase in dsRNA foci observed in EndoUmut-infected cells by immunofluorescence staining (Fig. 1).

**Fig. 3.**
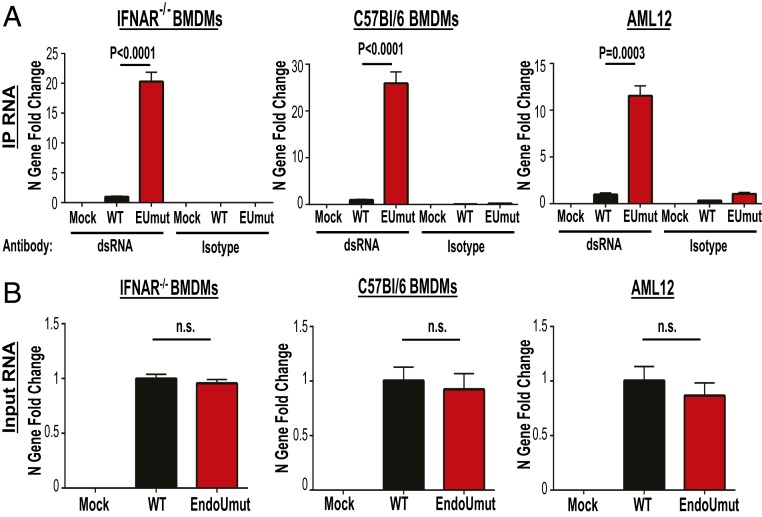
Quantifying viral RNA immunoprecipitated with antibody K1. IFNAR^−/−^ BMDMs, C57BL/6 BMDMs, and AML12 cells were infected with WT or EndoUmut virus (EUmut) at an MOI of 1. At 6 hpi, RNA was collected and processed for dsRNA immunoprecipitation (IP) with anti-dsRNA antibody or an isotype control. (*A*) CoV RNA immunoprecipitated using K1 Ab was quantified using primers to the nucleocapsid (N) gene by qPCR. (*B*) CoV RNA from input RNA was quantified by measuring the N gene expression. Viral RNA was normalized to 18s rRNA and set relative to WT. Values were analyzed by Student *t* tests. Data are representative of three independent experiments and presented as mean ± SD. n.s., not significant.

To determine the abundance of the dsRNA signal in other cell types, we infected IFNAR^−/−^ BMDMs, C57BL/6 BMDMs, and AML12 cells with either wild-type or EndoUmut virus, and performed the anti-dsRNA immunoprecipitation experiment. We used random hexamers as primers for complementary DNA (cDNA) synthesis, which allows for generation of cDNA from both positive- and negative-sense RNA, and then evaluated the abundance of cDNA by qPCR. We consistently detected elevated levels of viral RNA immunoprecipitated by the dsRNA antibody from EndoUmut virus-infected cells as compared to the levels detected in wild-type virus-infected cells (Fig. 3*A*). The total input viral RNA was similar between wild-type− and EndoUmut-infected cells (Fig. 3*B*). Overall, our sequencing and qPCR results suggest that EndoU reduces the accumulation of a negative-sense viral RNA epitope that can be recognized by the anti-dsRNA antibody.

### EndoU Activity Limits Abundance and Length of PUN RNAs.

**Fig. 4.**
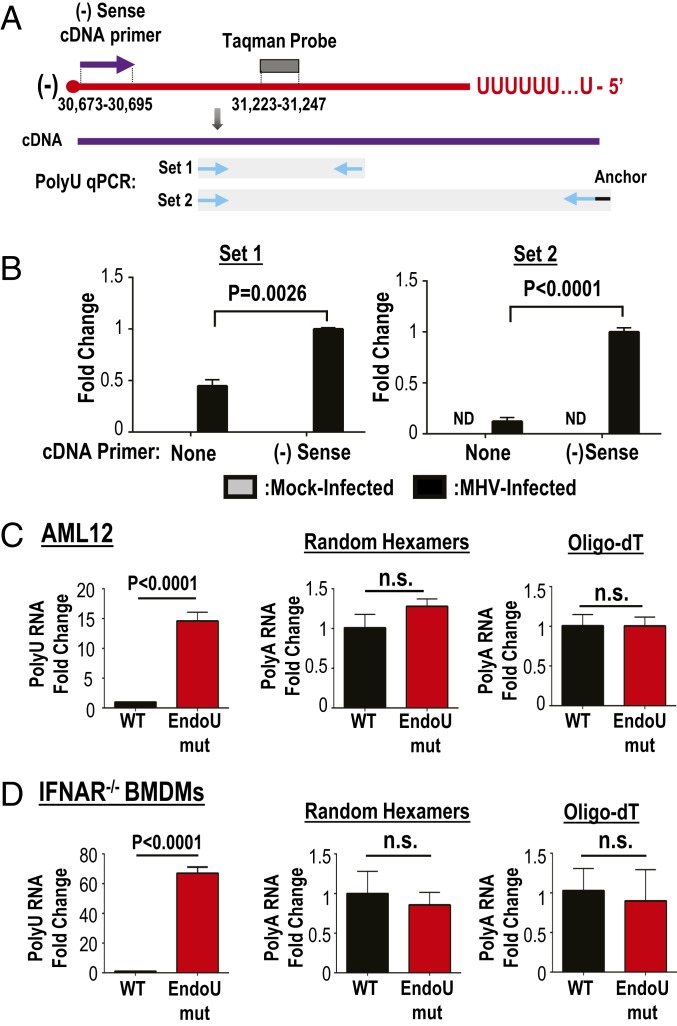
Quantifying PUN RNAs from virus-infected cells. IFNAR^−/−^ BMDMs and AML12 cells were infected with WT or EndoUmut virus at an MOI of 1, and RNA was purified from cell lysates. (*A*) Schematic of cDNA and qPCR design. The cDNA was generated using cDNA primers specific to the negative-sense RNA, random hexamers for total RNA, or oligo-dT primers for positive-sense RNA. The qPCR was performed with either primer set 1 or primer set 2 for each polyU qPCR. Nucleotide number where negative-sense (−) cDNA primer and probe bind to viral RNA are labeled. (*B*) The qPCR of cDNA synthesized with no primers or negative-sense cDNA primers. (*C*) PolyU qPCR of negative-sense RNA (*Left*) or PolyA qPCR primed with random hexamers (*Middle*) or oligo-dT primers (*Right*) from AML12 cells at 8 hpi. (*D*) PolyU qPCR of negative-sense RNA (*Left*) or PolyA qPCR primed with random hexamers (*Middle*) or oligo-dT primers (*Right*) from IFNAR^−/−^ BMDMs at 6 hpi. Set 2 is normalized to set 1 and is presented as mean ± SD. Values were analyzed by Student *t* tests. Data are representative of three independent experiments. ND, not detected; n.s., not significant.

Previous studies showed that the 5′ end of the CoV negative-sense RNA contains polyU extensions (35), and that EndoU cleaves at uridine residues (22, 25, 27–30). Therefore, we considered the PUN RNA as a potential target for EndoU activity. We hypothesized that PUN RNAs accumulate in the absence of EndoU activity. To quantitate the PUN RNAs, we generated cDNA from the negative-sense RNA using a strand-specific primer and performed a series of qPCRs with primers shown in Fig. 4*A*. Primer set 1 flanks a taqman probe and provides a measurement of total negative-sense RNA. Primer set 2 measures the PUN RNA. By normalizing set 2 to set 1, we can compare relative proportions of the negative-sense RNA that contain polyU sequences. To control for potential “self-priming” of the viral RNA during cDNA synthesis, we performed cDNA synthesis in the presence or absence of the negative-sense cDNA primer and quantified RNA expression by qPCR (Fig. 4*B*). For both set 1 and set 2 qPCRs, we detected a significantly higher signal with the negative-sense primer compared to no primer. When comparing wild-type− and EndoUmut-infected cells, we detected a 10-fold increase in PUN RNAs from EndoUmut-infected cells as compared to wild-type virus-infected AML12 cells (Fig. 4*C*, *Left*) and detected a 60-fold increase in IFNAR^−/−^ BMDMs (Fig. 4*D*, *Left*). To determine whether the polyA tail on the positive-sense RNA was similarly reduced by EndoU activity, we used either random hexamers or oligo-dT primers for reverse transcription and determined that the abundance of polyA tails on positive-sense RNA does not differ between wild-type and EndoUmut infections (Fig. 4 *C* and *D*, *Middle* and *Right*). We concluded that EndoU activity reduces the abundance of negative-sense RNA that contains polyU extensions.

**Fig. 5.**
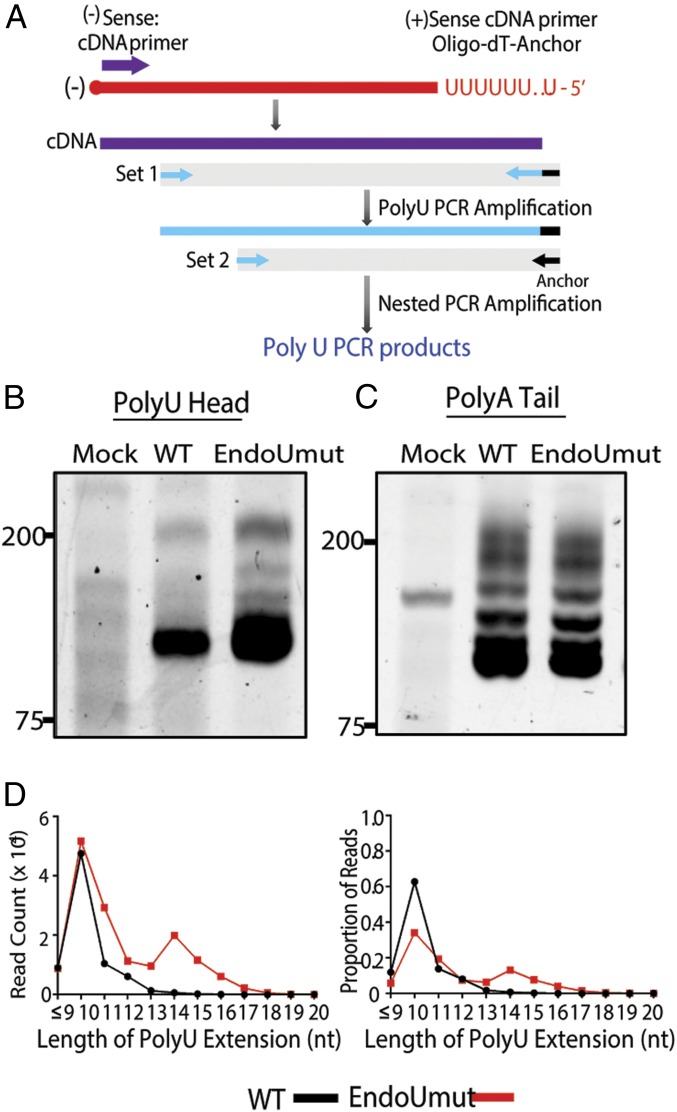
Evaluating the length of polyU extensions on PUN RNA. AML12 cells were infected with WT or EndoUmut virus at an MOI of 1. At 8 hpi, RNA was purified from cell lysates, and polyU nested PCR was performed. (*A*) Schematic of nested PCR. Briefly, cDNA was generated with a strand-specific primer for negative-sense (−) RNA or an oligo-dT anchor primer for positive-sense RNA, and then nested PCR was performed. (*B*) PolyU or (*C*) PolyA PCR products separated on a 10% polyacrylamide gel and stained with SYBR Green II. (*D*) PolyU PCR products were purified from the polyacrylamide gel in *B* and sequenced with MiSeq Next-Gen Sequencing. Graph of read counts that contain a specific nucleotide (nt) length of polyU extensions (*Left*). Graph of proportion of reads that contain a specific length of polyU extensions (*Right*). Data are representative of three independent experiments.

To determine whether EndoU reduces the lengths of the polyU extensions on the PUN RNA, we completed a nested PCR to obtain polyU-containing PCR products with a minimum predicted size of ∼100 base pairs (bp) (Fig. 5*A*). We detected PCR species of ∼100 bp from both wild-type− and EndoUmut-infected cells, and detected a smear of larger PCR species unique to EndoUmut virus-infected cells (Fig. 5*B*). To determine whether the length of polyA tails on the positive-sense RNA was affected by EndoU activity, we generated cDNA with oligo-dT primers to select for polyA-containing RNAs and performed the nested PCR reactions. We found that the products generated from positive-sense RNA were similar between wild-type and EndoUmut viruses, consistent with our previous results indicating that the polyA tail is not cleaved by EndoU activity (Fig. 5*C*). To determine whether the smear of PCR amplicons represents extended polyU sequences, we sequenced the amplicons with next-generation sequencing and found that EndoUmut PCR amplicons had an increase in the number of reads and proportion of products with extended polyU sequences (Fig. 5*D*). The most striking feature of the sequencing results is the bimodal distribution of the polyU extensions present in the EndoUmut-infected cell samples. We found that the majority (65%) of the reads from wild-type virus infection contained 10 uridine residues. In contrast, only 35% of the reads from the EndoUmut virus-infected sample contained 10 uridine residues. This was not due to a difference in the number of reads with 10 uridines but to an increase in longer polyU extensions detected in EndoUmut-infected cells. We detected variability in the polyU extensions in the EndoUmut virus-infected sample, with 65% of the reads containing from 11 to 17 uridine residues. We note that, while PUN RNAs in EndoUmut-infected cells are only a few uridines longer than the PUN RNAs from wild-type virus-infected cells, the PUN RNAs are 10-fold more abundant in EndoUmut virus-infected cells (Fig. 4). Overall, these experiments revealed that EndoU activity reduces the abundance and length of polyU extensions on PUN RNAs, consistent with our hypothesis that EndoU cleaves the PUN RNAs during virus replication.

**Fig. 6.**
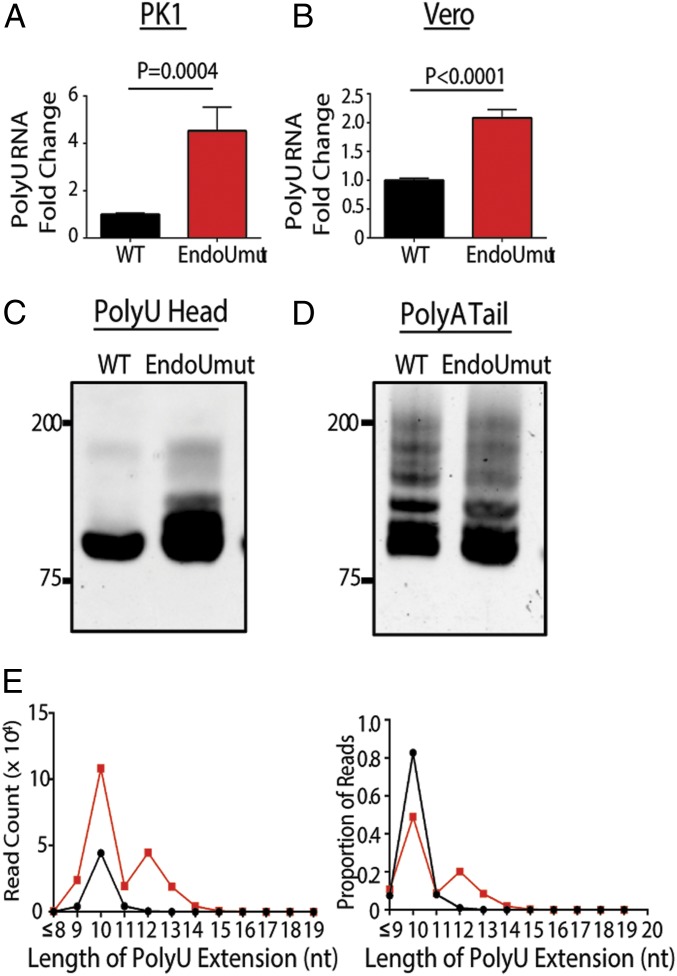
Evaluating the abundance and length of PUN RNA during PEDV infection. PK1 or Vero cells were infected with WT or EndoUmut PEDV at an MOI of 0.1. RNA was purified at 24 hpi. (*A*) PolyU qPCR quantified in PK1 cells. (*B*) PolyU qPCR quantified in Vero cells. Set 2 is normalized to set 1 and is presented as mean ± SD. (*C*) PolyU or (*D*) PolyA nested PCR products from PK1 Cells. (*E*) PolyU PCR products from PK1 cells were purified from the polyacrylamide gel in *C* and sequenced with MiSeq Next-Gen Sequencing. Graph of read counts that contain a specific nucleotide (nt) length of polyU extensions (*Left*). Graph of proportion of reads that contain a specific length of polyU extensions (*Right*). Values were analyzed by a Student *t* test. Data are representative of two independent experiments.

Since EndoU is conserved among CoVs, we sought to determine whether EndoU reduces the abundance and length of the PUN RNAs in the alpha-CoV porcine epidemic diarrhea virus (PEDV). Although the EndoU domains of MHV and PEDV exhibit only about 50% overall amino acid similarity, the catalytic histidines are 100% conserved (19). We showed that inactivation of EndoU in PEDV results in an increased type I and type III IFN response during infection (20). To determine whether EndoU limits the accumulation of PUN RNAs during PEDV infection, we infected cells with either wild-type or EndoUmut PEDV, isolated RNA, and evaluated the levels of PUN RNAs. We found that, relative to wild-type virus-infected cells, EndoUmut virus-infected cells contained abundant PUN RNAs in PK1 (Fig. 6*A*) and Vero cells (Fig. 6*B*). Sequences of PCR products templated by PUN RNA revealed that the length of the polyU extensions on the PUN RNAs was increased during EndoUmut virus infection (Fig. 6 *C* and *E*), with a similar bimodal distribution of polyU extensions shown in Fig. 5*D*. During PEDV infection, we did not observe a difference in polyA tail length (Fig. 6*D*). Taken together, these results indicate that PUN RNAs are generated during alpha- and beta-CoV replication, and that the highly conserved EndoU activity targets the polyU extensions in the PUN RNA.

### PUN RNA Is a PAMP.

**Fig. 7.**
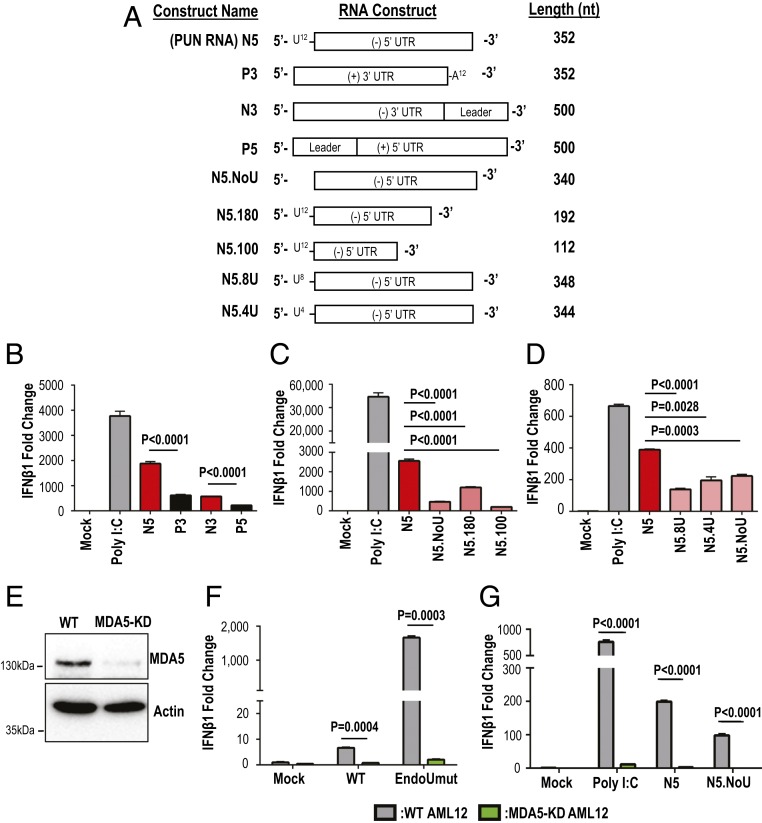
Determining whether PUN RNA is an MDA5-dependent PAMP. RNA was in vitro-transcribed from DNA constructs and transfected into AML12 cells. At 8 hpt, RNA was purified from cell lysates, and IFNβ1 gene expression was measured by qPCR. (*A*) Schematic diagram of RNA products from in vitro transcription. (*B*) IFNβ1 gene expression induced by coronaviral RNA termini. (*C*) IFNβ1 gene expression by PUN RNA constructs. (*D*) IFNβ1 gene expression by PUN RNA constructs with varying polyU lengths. MDA5-knockdown AML12 cells (MDA5-KD) were generated by CRISPR/Cas9 transduction. (*E*) Western blot of WT and MDA5-KD AML12 cells for MDA5 and Actin. (*F*) WT and MDA5-KD AML12 cells were infected with WT or EndoUmut MHV at an MOI of 1. IFNβ1 expression was measured at 16 hpi. (*G*) In vitro transcribed PUN RNA was transfected into WT or MDA5-KD AML12 cells. IFNβ1 expression was measured at 8 hpt. IFNβ1 gene expression is normalized to 18s rRNA and set relative to mock. Values were analyzed by Student *t* tests. Data are representative of three independent experiments and presented as mean ± SD.

Since EndoU both reduces PUN RNA abundance and suppresses host MDA5 activation, we hypothesized that CoV PUN RNA is a PAMP. To directly test this hypothesis, we measured IFN stimulation following introduction of PUN RNAs derived from MHV-A59 into AML12 cells. PUN RNA was synthesized by T7 in vitro transcription of digested plasmids that contained sequences representing the 5′ end or 3′ end of the viral genome (Fig. 7*A*). The PUN RNA, designated N5, and other CoV positive- and negative-sense RNA termini (P3, P5, N3) were transfected into AML12 cells. Total cellular RNA was harvested at 8 h posttransfection (hpt) and subjected to qPCR for IFNβ1 messenger RNA (mRNA) expression. We found that the presence of PUN RNAs increased IFNβ1 expression by 2,000-fold (Fig. 7*B*), which was fourfold higher than any other in vitro transcribed viral RNA, indicating that PUN RNA is a PAMP.

To determine whether the polyU sequence contributed to the robust IFN stimulation of the PUN RNA, we transcribed PUN RNA containing either 12 uridines (N5) or no uridines (N5.NoU) at the 5′ end. We found that removing the 12 uridines from the PUN RNA significantly decreased the ability of that RNA to induce IFNβ1 expression (Fig. 7*C*). Also, removing sections of the 3′ end of the PUN viral sequence (N5.180 and N5.100) resulted in a decrease in IFNβ1 expression, suggesting the polyU sequence alone is not sufficient to induce the IFN response (Fig. 7*C*). Shortening the polyU extension to eight uridines (N5.8U) or four uridines (N5.4U) also diminished the IFN activation by the PUN RNA (Fig. 7*D*). These results suggest that a polyU sequence of 12 uridines can enhance the IFN response to PUN RNA.

Previous studies documented that MHV-A59 infection induces IFN through MDA5 signaling (6, 11). To determine whether PUN RNA activates MDA5, we generated MDA5 knockdown (MDA5-KD) AML12 cells by CRISPR-Cas9 transduction (Fig. 7*E*) and measured IFN activation by virus infection or RNA transfection. Both viral infection and the transfection of the PUN RNA induce IFNβ1 expression in an MDA5-dependent manner (Fig. 7 *F* and *G*). During viral infection of MDA5-KD cells, both wild-type and EndoUmut virus infections had a significant reduction of IFNβ1 expression (Fig. 7*F*). IFNβ1 induction by in vitro transcribed PUN RNA also was significantly reduced in MDA5-KD cells (Fig. 7*G*). Importantly, we found that a single-stranded, in vitro-transcribed RNA activated MDA5, which was previously known to be activated by long complementary dsRNA. Taken together, these data suggest that the PUN RNA can act as an MDA5-dependent, viral PAMP.

### EndoU Can Degrade PUN RNA and Dampen IFN Activation.

**Fig. 8.**
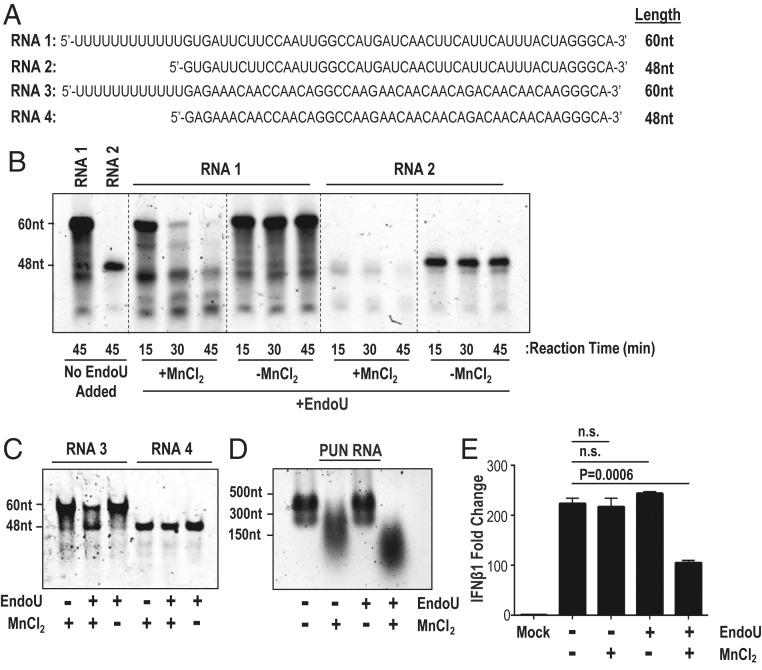
Evaluating EndoU activity on PUN RNA. RNA was cleaved by EndoU and separated by gel electrophoresis. (*A*) Sequences and length of RNAs 1 to 4. (*B*) EndoU cleavage of RNA 1 and RNA 2 performed for stated times and separated on a 10% polyacrylamide gel. (*C*) EndoU cleavage of RNA 3 and RNA 4 for 30 min and separated on a 10% polyacrylamide gel. (*D*) EndoU cleavage of in vitro transcribed PUN RNA (N5) for 45 min and separated on a 1% agarose gel. (*E*) RNA treated by EndoU cleavage was transfected in AML12 cells, and, at 8 hpt, RNA was purified from cell lysates, and IFNβ1 gene expression was measured by qPCR. IFNβ1 gene expression is normalized to 18s rRNA and set relative to mock. Values were analyzed by Student *t* tests. Data are representative of three independent experiments and presented as mean ± SD. n.s., not significant.

To determine whether EndoU activity can cleave the PUN RNA PAMP, we performed a series of in vitro cleavage assays (29). We incubated EndoU with 5′ negative-sense RNA containing a 12-uridine extension (RNA 1) or without the 12-uridine extension (RNA 2) (Fig. 8*A*). When either RNA 1 or RNA 2 is mixed with EndoU in the presence of MnCl_2_, the RNA is degraded over time (Fig. 8*B*). This degradation is most likely due to the presence of multiple uridines throughout RNA 1 and RNA 2, which is consistent with previous studies (22). We observed that EndoU cleaves RNA 1 more slowly than RNA 2 in this assay. We speculate that the polyU extension on RNA 1 may promote the formation of RNA secondary structures, which could contribute to the relative stability of RNA 1 versus RNA 2.

To determine whether the polyU extension can be cleaved, we substituted the viral sequence uridines with adenosines and generated RNA 3 and RNA 4 (Fig. 8*A*). When mixed with EndoU and MnCl_2_, the polyU extension of RNA 3 is cleaved, producing a cleavage product the size of RNA 4 (Fig. 8*C*). RNA 4 was not cleaved, consistent with the requirement of uridine residues for EndoU recognition and cleavage. To determine whether EndoU cleavage can decrease the ability of PUN RNA to stimulate IFN, we cleaved the PUN RNA with EndoU (Fig. 8*D*). In the presence of EndoU and MnCl_2_, the PUN RNA was degraded into smaller RNA fragments. After EndoU treatment, we transfected the PUN RNAs into AML12 cells and measured IFN stimulation (Fig. 8*E*). We found that transfecting the RNA treated with EndoU decreased the IFN stimulation activity. We note that the PUN RNA with MnCl_2_ migrated faster in the agarose gel, likely due to the addition of the Mn^2+^ cation (36), but we do not observe a difference in IFN stimulation in the presence of MnCl_2_ alone. Overall, EndoU is capable of cleaving and degrading PUN RNA, which then reduces the ability of PUN RNA to stimulate IFN.

## Discussion

Our study reveals that CoV endoribonuclease activity degrades PUN RNA, which acts as a viral PAMP. EndoU cleaves the polyU sequence on the PUN RNA, limiting the length and abundance of the polyU extension. This reduces the IFN-stimulating effect of PUN RNA, which, without EndoU digestion, activates host sensor MDA5. The fact that EndoU is highly conserved in all CoVs suggests that EndoU activity is important for sustained replication in the host (19, 37). Our study reveals that the PUN RNA is a PAMP and that EndoU activity is essential for limiting the accumulation of PUN RNA.

**Fig. 9.**
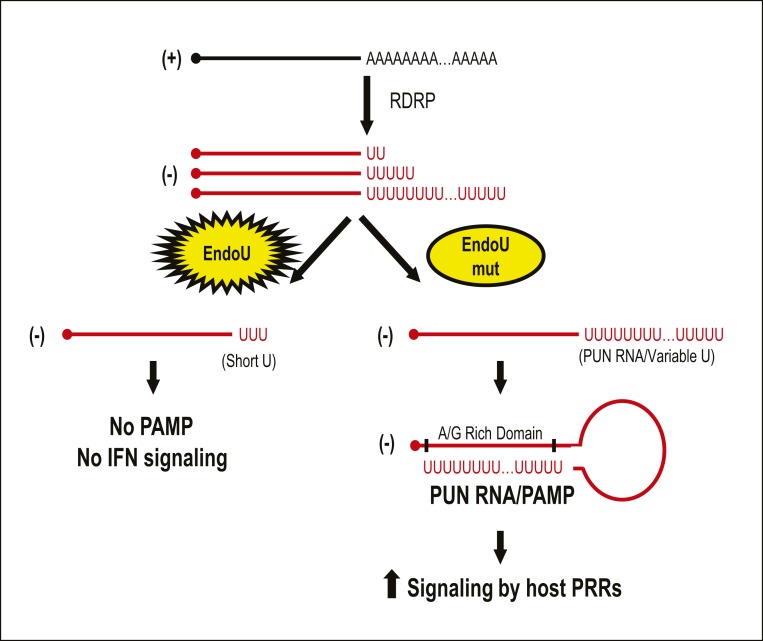
Model depicting EndoU cleavage of PUN RNAs. This model depicts how EndoU activity limits the generation of PUN RNA, which can act as a PAMP. We found that PUN RNAs with variable lengths of polyU sequences are generated in the absence of EndoU activity. We predict that these PUN RNAs can fold back and generate stem−loop structures by hybridizing with an A/G-rich domain located within the PUN RNA or on adjacent RNAs. This stem−loop structure may be recognized as dsRNA by host PRRs, thus stimulating the host innate immune response. The function of EndoU during replication is to reduce the length of polyU sequences, thus limiting the potential for generating PAMPs.

We developed a model consistent with our findings (Fig. 9). We hypothesize that, during the synthesis of negative-sense RNA, the CoV RNA-dependent RNA polymerase uses the polyA tail as a template to generate negative-sense RNAs with variable lengths of polyU extensions. EndoU can recognize and cleave the polyU extensions, which limits the ability of the negative-sense RNA to form a viral PAMP. In the absence of EndoU activity, the polyU extension on the PUN RNA enhances the interactions of the PUN RNA with a complementary region of the viral genome to form an epitope recognized by MDA5 and the K1 anti-dsRNA antibody.

One of the surprising findings from our study is that antibody K1, which was developed as an anti-dsRNA antibody (38), recognizes CoV negative-sense RNA (Figs. 1 and 2). Our immunofluorescence studies showed that the epitope recognized by K1 accumulates in EndoUmut-infected cells. Using RNA sequencing (RNA-seq), we determined that the RNA bound by K1 was negative-sense RNA. We speculate that CoV negative-sense RNA forms a higher-order RNA structure recognized by the K1 antibody, and that this RNA is also recognized by host sensors. Supporting this idea, a previous study showed that the viral RNA recognized by the K1 antibody during encephalomyocarditis virus infection formed a higher-order RNA structure and could activate MDA5 (39). Our approach using RNA-seq analysis of immunoprecipitated RNA could be widely used to determine whether other unique dsRNA epitopes are generated during viral infections. Schönborn et al. developed four anti-dsRNA antibodies: J2, J5, K1, and K2 (38). These antibodies were generated against the L species of dsRNA from *Saccharomyces cerevisiae*, and each antibody has unique binding specificities to different dsRNA species. For example, the K1 antibody was reported to be highly specific to poly I:C, whereas the J2 antibody is specific to the L species of dsRNA (38, 40). The differing specificities suggest that each anti-dsRNA antibody recognizes unique dsRNA structures or sequences. Ultimately, structural studies are needed to fully elucidate the higher-order RNA structures that these dsRNA antibodies are recognizing during CoV infection.

Identifying viral PAMPs and the host PRRs that are activated by the PAMPs is critical for developing strategies for treating viral infections. Previous studies implicated MDA5 as the PRR important for macrophages to respond to CoV infection (6, 18). Consistent with these studies, we report that the PUN RNA acts as a PAMP, recognized by MDA5 (Fig. 7). We show that knocking down MDA5 by CRISPR-Cas9 limits the IFN stimulation by PUN RNA. Canonically, MDA5 binds to long dsRNA species, such as poly I:C, to induce IFN signaling (4). Our study suggests that the negative-sense RNA may form a higher-order RNA structure that can bind and activate MDA5. Interestingly, a longer polyU extension (>12 uridines) on the RNA drives a heightened IFN response. Currently, many studies utilize nucleic acid as an adjuvant to stimulate innate immune responses (41). With the PUN RNA forming an MDA5-recognized structure, it would be interesting to determine whether the PUN RNA could act as an MDA5 adjuvant to elicit robust IFN responses during immunizations.

Our study raises the question of whether other viruses have mechanisms to limit polyU-containing RNA from activating host PRRs. The hepatitis C virus (HCV) genome encodes a long polyU stretch on the positive-sense RNA, but the polyU region is flanked by highly structured RNA, which may limit immune stimulation (42). Furthermore, the HCV replication complexes may hide the viral RNA from recognition by host sensors (43). Polioviruses prime replication of the negative-sense RNA with a polyU sequence attached to VPg (44). The VPg linkage may prevent the exposure of a polyU structure to host sensors, thus preventing the polyU sequence from acting as a PAMP. For influenza viruses, the polyU sequence on negative-sense RNA is essential for polyadenylation, because it is part of a unique stem−loop structure (45). However, this RNA structure is localized to the nucleus and not exposed to cytoplasmic dsRNA sensors. These examples illustrate that many viruses have polyU sequences that may act as PAMPs, and that each virus may have evolved unique mechanisms or structures that limit their detection.

Our study also raises interesting questions about how the CoV polyA tail is generated during positive-sense RNA synthesis. Studies from influenza virus revealed that a short polyU sequence within a unique RNA stem−loop mediates a stuttering mechanism used to polyadenylate the positive-sense RNA (45). In contrast, Peng et al. (46) implicate that CoVs utilize a noncanonical cytoplasmic polyadenylation site to synthesize the positive-sense polyA tail. They identified a conserved viral sequence on the positive-sense RNA that could be bound by host proteins, including cytoplasmic polyadenylation element binding protein 1 (CPEB1) (47), a protein that mediates polyadenylation. In addition, CoV nsp8 has been demonstrated to contain 3′ terminal adenylyltransferase (TAT) activity (48). Nsp8 can synthesize the polyA tail on the positive-sense RNA, and having a complement negative-sense RNA with a polyU extension greater than five uridines enhances the TAT activity. Since we observe EndoU controlling the abundance of longer polyU sequences which stimulate IFN, it would be interesting to determine whether there is an “ideal length” of polyU sequences that lack immune stimulation by MDA5, but promote TAT activity during addition of the polyA tail. One caveat of these in vitro studies is that the assays are performed in the absence of other viral replication complex proteins that may alter the binding, recognition, and activity of the RNA processing proteins. Studies have shown protein−protein interactions between nsp12, nsp7, nsp8, and nsp15, which may alter the activities of these proteins (31, 49). While EndoU can fully degrade PUN RNAs in vitro, the cleavage activity may be more specific during viral infection, due to interactions with other viral proteins in the membrane-associated replicase complex (50, 51). While EndoU can cleave PUN RNA, there may also be other EndoU cleavage sites during CoV infection that were not detected in this study. Further studies are needed to fully elucidate the mechanisms CoVs use to alter and process viral RNAs.

Our study reveals that the CoV endoribonuclease activity is distinctly different from three other previously documented viral ribonucleases. Influenza PA-X is an endoribonuclease that selectively degrades host mRNA by hijacking host RNA splicing machinery (52). PA-X inhibits the translation of host proteins to perturb the cell functions. Pestivirus RNase E(rns) is an endoribonuclease that is secreted outside infected cells and degrades extracellular viral RNAs to block innate immune activation (53). Lassa virus encodes an exonuclease that will specifically degrade intracellular dsRNA (54). Both RNase E(rns) and Lassa virus exonuclease cleave viral RNAs thought to be PAMPs. Our study reveals an additional mechanism for a viral endoribonuclease to degrade a viral PAMP.

The current outbreak of 2019-nCoV and the associated morbidity and 2% mortality highlight the importance of developing effective vaccines against CoVs (55–57). One vaccine strategy is to generate attenuated viruses that can efficiently be produced and signal for robust, protective, antiviral immune responses. EndoU is not necessary for viral replication, and EndoUmut CoVs are attenuated in vivo while still eliciting a protective immune response (11, 20). Therefore, EndoU may be one of several immune antagonists targeted for generating an attenuated and recombination-resistant CoV vaccine (58, 59). Ideally the IFN antagonist mutations would be conserved so they can be applied to any current or emergent CoV, including the 2019-nCoV. In addition, the enzymatic activity of EndoU could be targeted for the development of an antiviral therapeutic.

In summary, this study provides evidence for a mechanism used by the CoV EndoU to cleave a viral RNA PAMP, which would otherwise be recognized by MDA5. EndoU activity delays recognition by the host innate immune sensors, and thus is a highly conserved virulence factor and a potential target for antiviral and vaccine strategies.

## Materials and Methods

### Cells, Viruses, and Reagents.

AML12 hepatocytes (CRL-2254; ATCC) were cultured in Dulbecco’s modified Eagle’s medium (DMEM)/F-12 (12400-024, Invitrogen) supplemented with 1% penicillin/streptomycin, 10% fetal bovine serum (FBS), Insulin, Transferrin, and Selenium (41400045; Life Technologies), and Dexamethasone (40 ng/mL, D4902; Sigma). L929 cell line was gifted from Francis Alonzo, Loyola University of Chicago, Maywood, IL, and maintained in DMEM (10-017-CV; Corning) supplemented with 10% FBS, 1% l-glutamine, 1% sodium pyruvate, 1% nonessential amino acids, and 1% penicillin/streptomycin. Differentiated BMDMs were maintained in bone marrow macrophage media containing DMEM (10-017-CV; Corning) supplemented with 30% L929 cell supernatant, 20% FBS, 1% l-glutamine, 1% sodium pyruvate, and 1% penicillin/streptomycin. Methods for generation of BMDMs are described in Deng et al. (11). Porcine kidney epithelial cells, PK1 (CL101; ATCC), were grown in growth medium containing modified Eagle medium (MEM) (10-010-CV; Corning) supplemented with 5% FBS, and 1% penicillin/streptomycin. Vero cells were grown in growth media containing MEM (41500-018; Gibco) supplemented with 10% FBS, 0.5% lactalbumin enzymatic hydrolysate (68458-87-7; Sigma), and 1% penicillin/streptomycin. Wild-type MHV strain A59 (GenBank accession no. AY910861) and EndoUmut (H262A) were previously generated by reverse genetics and full-genome sequenced (11). Infectious clones of wild-type PEDV or EndoUmut PEDV (H226A) were previously generated by reverse genetics and full-genome sequenced (20).

### dsRNA Immunoprecipitation PCRs.

IFNAR^−/−^ BMDMs, C57BL/6 BMDMs, or AML12 cells were infected with wild-type or EndoUmut virus at a multiplicity of infection (MOI) of 1. At indicated times postinfection, RNA was isolated with RNeasy kit (74104; Qiagen). One microgram of RNA was mixed with dsRNA antibody (K1; Scicons) or mouse anti-Beta actin (A00702-40; Genscript), RNA binding buffer (50 mM Tris pH 8.0, 150 mM NaCl, 1% Nonidet P-40, and 20 U/mL Ribolock [EO0381; Thermofisher]). After overnight incubation, protein G beads (LSKMAGG02; Millipore) were added and incubated rotating for 4 h at 4 °C. Protein G beads were precipitated with magnets, then washed with cold binding buffer. RNA was purified off beads with RNeasy Kit and reverse-transcribed into cDNA with RT2 First Strand kit (330411; Qiagen). The qPCR was performed as described below.

For RNA-seq, RNA was processed by University of Chicago Genomics Facility. The cDNA sequencing libraries were generated with TruSeq Stranded Total RNA with Ribo-zero extraction (Illumina), then sequenced on Illumina HiSeq4000. RNA-seq reads were analyzed with Galaxy’s online platform (https://usegalaxy.org/). Reads were groomed, clipped, and mapped with Hisat2 to the wild-type MHV strain A59 (GenBank accession no. AY910861) or host genome (GRCm38 Ensembl build of the C57BL/6J). The number of reads at individual nucleotides was calculated by plotCoverage. RNA-seq data have been deposited and are available in the National Center for Biotechnology Information Gene Expression Omnibus (NCBI GEO) database (accession no. GSE144886).

### qPCR.

RNA was isolated with RNeasy kit from cells at stated times postinfection. Isolated RNA was reverse-transcribed with RT2 First Strand Kit. The qPCR reactions were performed using RT2 Kit (330502; Qiagen). The qPCR reaction is 1) 95 °C for 10 min, 2) 95 °C for 15 s, 3) 60 °C for 1 min, and a repeat of steps 2 and 3 for 40 cycles.

### PolyU Extension PCRs.

Cells were infected with wild-type or EndoUmut virus at an MOI of 1. At indicated times postinfection, RNA was isolated with RNeasy Kit. For strand-specific cDNA, 500 ng of RNA was reverse-transcribed with Omniscript (205113; Qiagen) with a reaction temperature of 50 °C to reduce self-primed cDNA synthesis (60). For negative-sense RNA, the cDNA primer was 5′-GAATTCTGGTGGTGCTGATGAAC-3′ for MHV and 5′-GCAGCATTGCTCTTTGGTG-3′ for PEDV. For total RNA, the cDNA primers were random hexamers, and positive-sense RNA primer was oligo-dT. The qPCR was performed with SsoAdvanced Universal Probes Supermix (1725281; Bio-Rad). The qPCR reaction was performed with an annealing temperature of 60 °C with either primer set PolyU qPCR set 1 or PolyU qPCR set 2. Primers are listed in *SI Appendix*, Table S1.

PolyU length nested PCRs were performed with PFU Ultra Polymerase (600380; Agilent) with an annealing temperature of 60 °C. The cDNA was generated as described above. For negative-sense RNA, the cDNA primer was 5′-GAATTCTGGTGGTGCTGATGAAC-3′ for MHV and 5′-GCAGCATTGCTCTTTGGTG-3′ for PEDV. For positive-sense RNA, the cDNA primer was 5′-GGGGATCCGCGGTTTTTTTTTT-3′. A PCR was performed with set 1 primers for PolyU length. Then the product was diluted 1:1,000 and utilized in a PCR with set 2 primers for PolyU length. PCR products were separated on a 10% polyacrylamide gel and stained with SYBR Green II dye (S7564; Thermofisher). Primers for cDNA synthesis and PCRs are listed in *SI Appendix*, Table S1. For polyU length sequencing, sequencing libraries were generated with custom amplicon primers with nextera XT indexes, and the amplicons were sequenced on an Illumina Miseq V2 500 with paired-end 250-bp reads. Reads were analyzed and mapped to viral genomes with Galaxy’s online platform.

### RNA Transfections.

The pCAGGs constructs were generated containing a T7 promoter, the viral genome segment, and a HindIII cleavage site. Viral sequences are listed in *SI Appendix*, Table S2. Plasmids were digested with HindIII-HF (R3104; NEB), then purified with Wizard Gel and PCR Purification Kit (A9282; Promega). RNA was in vitro-transcribed using a T7 RNA polymerase (EP0111; Thermofisher) and purified by LiCl precipitation. Five picomoles of RNA or Poly I:C (P1530; Sigma) was transfected into AML12 cells using Lipofectamine 2000 (11668027; Thermofisher). At 8 hpt, RNA was isolated using RNeasy kit and qPCR for IFNβ1 (PPM03594C; Qiagen), and 18s ribosomal RNA (rRNA) (PPM57735E; Qiagen) was performed as described above.

### Generation of MDA5-Knockdown Cells.

A modified CRISPR/Cas9 protocol, based on the GeCKO system (61), was used to knock down the function of MDA5 in AML12 cells. Single-guide RNA was identified with Benchling (Benchling, Inc.) to target the *Ifih1* gene. Sequence used for targeting *Ifih1* was 5′-ATGGACGCAGATGTTCGTGG-3′. The cDNA versions of guide RNA were annealed and inserted into a pLentiCRISPRv2-puro (Addgene 52961) cassette between flanking BsmBI sites. Transducing particles (TPs) were generated by transfecting HEK-293T/17 cells with pLentiCRISPRv2-puro, pPax2, and pHEF-VSV-G and collecting supernatant. TPs were centrifuged at 1,000 × *g* for 10 min at 4 °C then filtered through a 0.45-μM filter (Millipore Sigma). AML12 cells were transduced with TPs, then incubated for 24 h at 37 °C in 5% CO_2_. Transduced AML12 cells were then selected with 1 μg/μL puromycin (InvivoGen) for 96 h. Puromycin-selected cells were then grown and cloned into a monoclonal population. Knockdown of MDA5 was determined by Western blot using rabbit anti-MDA5 (SAB3500356; Sigma) and mouse anti-actin (A00702-40; Genscript).

### RNA Cleavage Assay.

Cleavage of RNA substrates was performed according to Kang et al. (29). Purified, wild-type EndoU was kindly gifted by C. Kao, formerly of Indiana University, Bloomington, IN, currently at Aligos Therapeutics, San Francisco, CA. Briefly, 1 μM RNA was mixed with EndoU in Cleavage Buffer (50 mM Tris pH7.5, 50 mM KCl, 1 mM dithiothreitol) with or without 5 mM MnCl_2_. Reactions were incubated at 30 °C for indicated time, and reactions were stopped by addition of RNA Gel Loading Buffer (B0363S; NEB) and incubation at 95 °C for 5 min. Reaction products were immediately loaded into a 10% polyacrylamide gel with Tris-Borate-ethylenediaminetetraacetic acid (EDTA) buffer or a 1% agarose gel with Tris-Acetate-EDTA buffer, and bands were separated by electrophoresis. Gels were then stained with SYBR Green II dye and visualized with a ChemiDoc XRS+ imager (Bio-Rad), and processed with Image Lab software (Bio-Rad).

### Data Availability Statement.

RNA-seq data have been deposited and are available in the NCBI GEO database (accession number: GSE144886).

## Supplementary Material

Supplementary File
