# Supplementary material for: Coronavirus endoribonuclease targets viral polyuridine sequences to evade activating host sensors
Source: Proc Natl Acad Sci U S A. 2020 Mar 20;117(14):8094–103. doi: 10.1073/pnas.1921485117 (PMC7149396; doi:10.1073/pnas.1921485117)
Supplement: Supplementary File [file pnas.1921485117.sapp.pdf]

SI Materials and Methods

AML12 growth Kinetics Cells were plated in 24-wells plates and subsequently infected with indicated viral strains at a MOI of 1 in serum-free media. After 1 h incubation, inoculum was replaced with fresh, complete medium. Cells were then incubated at 37°C 5% CO_2. At indicated time points, cell culture supernatants were collected and stored at -80°C. Supernatants were diluted and utilized for plaque assay on DBT cells. Briefly, 5 x 10^5 DBT cells/well were plated into 6 well plate and subsequently infected with diluted supernatants. After removal of inoculum, cells were overlaid with MEM containing 2% FCS and 0.4% Noble Agar. Cells were incubated for 48 h, then fixed with 3.7% formaldehyde in PBS. Fixed cells were stained with 0.1% crystal violet solution and plaques of cleared cells were quantified. Titers were obtained from three independent assays for each sample. Gene expression was performed from intracellular RNA. RNA was isolated using RNeasy kit and cDNA was synthesized with RT2 First Strand Kit (330411, Qiagen). qPCR for N gene, IFNβ1 (PPM03594C, Qiagen), IFNλ (F: AGCTGCAGGCCTTCAAAAAG, R: TGGGAGTGAATGTGGCTCAG) and 18s rRNA (PPM57735E, Qiagen)

Immunofluorescence. AML12 hepatocytes (1.5 × 10^5 cells per well) were plated on a glass coverslip in a 24-well plate for 24 h and subsequently infected with wild-type or mutant virus at an MOI of 0.1 in serum-free media. At 8 hpi, the infected cells were fixed with 4% formaldehyde in 0.095 M Pipes buffer (P1851, Sigma), permeabilized with 0.1% Triton X-100 (T8787, Sigma) in PBS, and blocked with 5% normal goat serum (NGS). Primary and secondary antibodies were used as follows: anti-nsp2/3 (1:1,500, Schiller *et al* (58)), anti-dsRNA (1:500, Scicons, K1), donkey anti-rabbit IgG alexafluor 488 (1:1,000; A-21441, Invitrogen), and goat anti-mouse IgG Alexa Fluor 568 (1:1,000; A11004, Thermofisher). Nuclei were visualized with Hoescht 33342 (1:2,500; H1339, Life Technologies). Cells were imaged by collecting z-stack images with a Deltavision wide-field fluorescent microscope (Applied Precision, GE) equipped with a digital camera (CoolSNAP HQ, Photometrics). Images were taken with a 100× lens. Samples were excited with light generated by an Insight SSI solid-state illumination module (Applied Precision, GE) and deconvolved with SoftWoRx deconvolution software (Applied Precision, GE). All images were collected under identical acquisition conditions and processed using Imaris 7.6.4 (Bitplane).

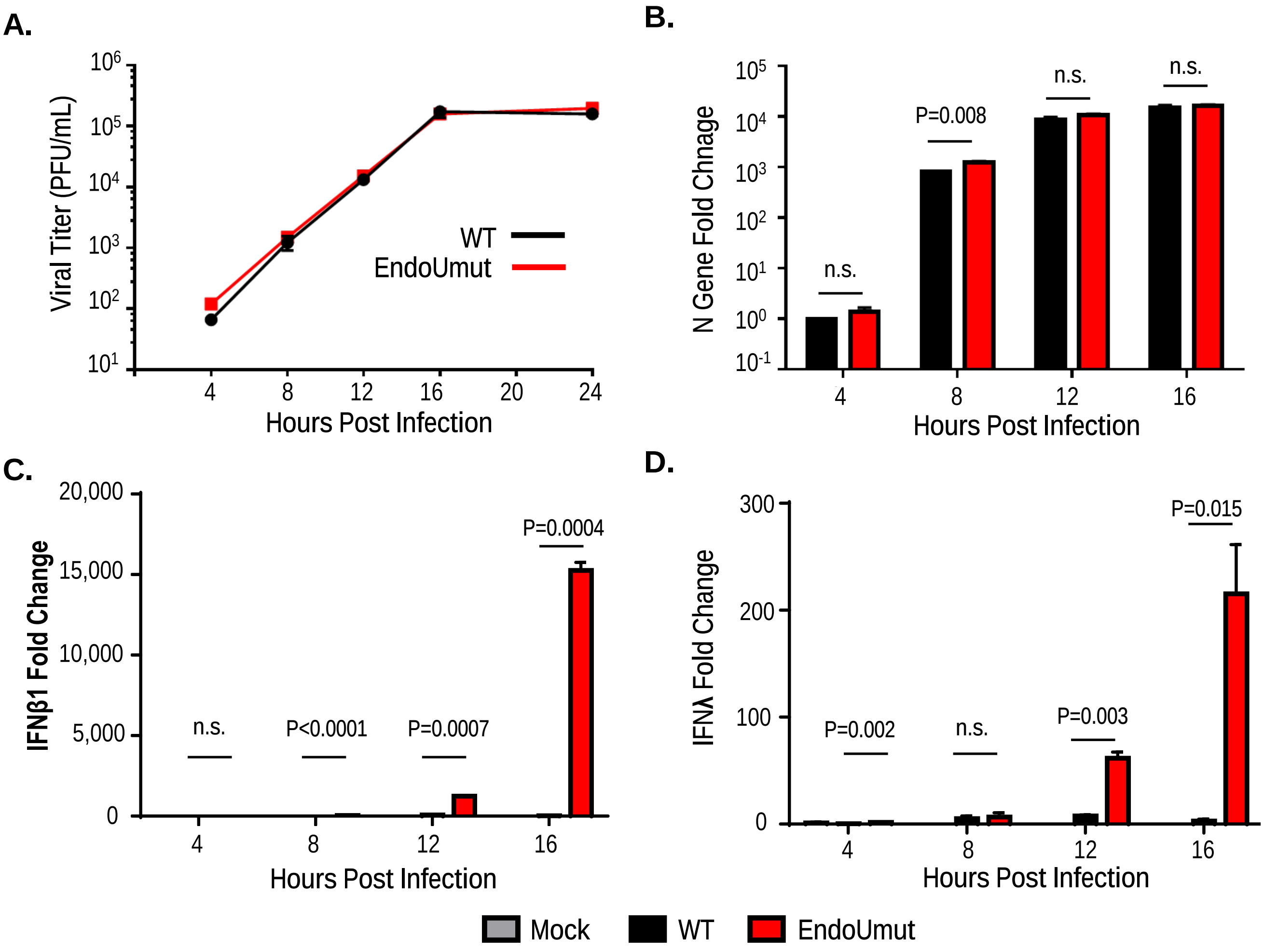

Figure S1. Growth Kinetic of wild-type and EndoUmut MHV-A59 in AML12 hepatocytes. AML12 cells were infected with wild-type (WT) or EndoUmut MHV-A59 at an MOI of 1. At indicated timepoints, supernatant and intracellular RNA was collected. (A) Growth kinetics of WT- and EndoUmut-viruses as measured by plaque assay in DBT cells. (B) Viral gene expression measured by qPCR of N gene transcripts. Gene expression is normalized to 18s rRNA and set relative to WT at 4hpi. (C) IFNβ1 gene expression as measured qPCR. (D) IFNλ gene expression as measured by qPCR. Gene expression is normalized to 18s rRNA and set relative to mock. Values were analyzed by student T-tests. Data are representative of three independent experiments and presented as mean ± standard deviation.

Table S1. Primers for PCR reactions.

Virus	Primer set		Sequence
MHV	Negative-Sense cDNA		5’-GAATTCTGGTGGTGCTGATGAAC-3’
	PolyU qPCR Set 1	R	5’-TGTGTGAGAGAAGTTAGCAAGG-3’
		F	5’-GCAGGAATAGTACCCTGATGTG-3’
	PolyU qPCR Set 2	R	5’-TGTGTGAGAGAAGTTAGCAAGG-3’
		F	5’-GGGGATCCGCGGTTTTTTTTTT-3’
	PolyU Length Set 1	R	5’-CAGATGTGGTGAGCCCAAAG-3’
		F	5’-GGGGATCCGCGGTTTTTTTTTT-3’
	PolyU Length Set 2	R	5’-CACATCAGGGTACTATTCCTGC-3’
		F	5’-GATAGGGGATCCGCGGT-3’
	Probe		5’-6-FAM-TAACCATAA/ZEN/GAACGGCGATAGGCGC-/Iowa Black FQ-3’
PEDV	Negative-sense cDNA		5'-GCAGCATTGCTCTTTGGTG-3'
	PolyU qPCR Set 1	R	5'-CACTGTCATGAGGGGAACG-3'
		F	5'-GTCCTTGAGTCAAATACCTGG-3'
	PolyU qPCR Set 2	R	5'-CACTGTCATGAGGGGAACG-3'
		F	5’-GGGGATCCGCGGTTTTTTTTTT-3’
	PolyU Length Set 1	R	5'-CAAGCGTGAAACCACGC-3'
		F	5’-GGGGATCCGCGGTTTTTTTTTT-3’
	PolyU Length Set 2	R	5'-CCAGGTATTTGACTCAAGGAC-3'
		F	5’-GATAGGGGATCCGCGGT-3’
	Probe		5’-6-FAM-CCTTTGCACGAGTAATCAAAGATCCGC-Iowa Black FQ-3’

Table S2. List of RNA constructs for in vitro transcription.

RNA construct	Gene Block Sequence
N5	UUUUUUUUUUUUGUGAUUCUUCCAAUUGGCCAUGAUCAACUUCAUUCAUUUACUAGGGCAUUGCAGG<br>AAUAGUACCCUGAUGUGAGCUCUUCCCAGGGGGCGCCUAUCGCCGUUCUUAUGGUUAGACGUAGGACC<br>UUGCUAACUUCUCUCACACAUUCUCUAUUUUGCAAUCUCUUUCAACUAAUUGAUACAGGGUCUGCCAC<br>AACCUUCUCUAUCUGUUAUGACAGCAAGACAUCCAUUCUGAUAGAGAGUGUCCUAUCCCGACUUUCUC<br>GCGAGGGGUUACCACCGAGCGCCGACAUAGGAUUCAUUCUCUUUACACAUUAGAGUCAUCUUCUAACC<br>CAUCUGGCACUAC
P3	GUAGUGCCAGAUGGGUUAGAAGAUGACUCUAAUGUGUAAAGAGAAUGAAUCCUAUGUCGGCGCUCGG<br>UGGUAACCCCUCGCGAGAAAGUCGGAUAGGACACUCUCUAUCAGAAUGGAUGUCUUGCUGUCAUAA<br>CAGAUAGAGAAGGUUGUGGCAGACCCUGUAUCAAUUAGUUGAAAGAGAUUGCAAAAUAGAGAAUGUG<br>UGAGAGAAGUUAGCAAGGUCCUACGUCUAACCAUAAGAACGGCGAUAGGCGCCCCCUGGGAAGAGCU<br>CACAUCAGGGUACUAUUCCUGCAAUGCCCUAGUAAAUGAAUGAAGUUGAUCAUGGCCAAUUGGAAGA<br>AUCACAAAAAAAAAAAAA
N3	UAACUGGCUCCAGCAUGGGGCCCUGUGCCAAGAUAGUAAAAAUACCAUCUGGGCAGUAAUUGCUUCU<br>GCUGCCCAUCAGGUGUUUUAAAAGAACGGCGGUUGUGUCUAUACCAAUAUCCCUUUUGCUCUGAAGC<br>GGGGAUUCCAUUGGCAAUAGGCACUCCUUGUCCUUCUGCAAACUGAAACUCCUUUCCCUUUUGGAACU<br>GGGUAAUGCCAGAAAACCAGGAGUAAUGGGGAACCACACUCCCGGAGUUGGGUUGAGUAGUUGCAGU<br>CUGCUUUGGCUGAUUCCUUCUGCCUCUAUUUUGAUUAUUUGGUCCACGCUCGGUUUGGUCAGCCCAAG<br>UGGUCUUCUUGAGGAUUCCAUUACCAGCGCGGUUUACAGAGGAGCUUCUGCCACCGGCAUUUUCUUGC<br>CCAGGAACAAAAGACAUCCUUAAAGUUUAGAUUAGAUUUAAACUACAAGAGUUUUAGAGUUGAGAGG<br>GUACGUACGGACGCCAAUCACUCUUAUC
P5	GAUAAGAGUGAUUGGCGUCCGUACGUACCCUCUCAACUCUAAAACUCUUGUAGUUUAAAUCUAAUCU<br>AAACUUUAAGGAUGUCUUUUGUUCCUGGGCAAGAAAAUGCCGGUGGCAGAAGCUCCUCUGUAAACCG<br>CGCUGGUAAUGGAAUCCUCAAGAAGACCACUUGGGCUGACCAAACCGAGCGUGGACCAAAUAAUCAA<br>AAUAGAGGCAGAAGGAAUCAGCCAAAGCAGACUGCAACUACUCAACCCAACUCCGGGAGUGUGGUUCC<br>CCAUUACUCCUGGUUUUCUGGCAUUACCCAGUUCCAAAAGGGAAAGGAGUUUCAGUUUGCAGAAGGA<br>CAAGGAGUGCCUAUUGCCAAUGGAAUCCCCGCUUCAGAGCAAAAGGGAUAUUGGUAUAGACACAACC<br>GCCGUUCUUUUAAAACACCUGAUGGGCAGCAGAAGCAAUUACUGCCCAGAUGGUAUUUUUACUAUCU<br>UGGCACAGGGCCCCAUGCUGGAGCCAGUUA
N5.NoU	GUGAUUCUUCCAAUUGGCCAUGAUCAACUUCAUUCAUUUACUAGGGCAUUGCAGGAAUAGUACCCUG<br>AUGUGAGCUCUUCCCAGGGGGCGCCUAUCGCCGUUCUUAUGGUUAGACGUAGGACCUUGCUAACUUCU<br>CUCACACAUUCUCUAUUUUGCAAUCUCUUUCAACUAAUUGAUACAGGGUCUGCCACAACCUUCUCUAU<br>CUGUUAUGACAGCAAGACAUCCAUUCUGAUAGAGAGUGUCCUAUCCCGACUUUCUCGCGAGGGGUUA<br>CCACCGAGCGCCGACAUAGGAUUCAUUCUCUUUACACAUUAGAGUCAUCUUCUAACCCAUCUGGCACU<br>AC
N5.180	UUUUUUUUUUUUGUGAUUCUUCCAAUUGGCCAUGAUCAACUUCAUUCAUUUACUAGGGCAUUGCAGG<br>AAUAGUACCCUGAUGUGAGCUCUUCCCAGGGGGCGCCUAUCGCCGUUCUUAUGGUUAGACGUAGGACC<br>UUGCUAACUUCUCUCACACAUUCUCUAUUUUGCAAUCUCUUUCAACUAAUUGAUACA
N5.100	UUUUUUUUUUUUGUGAUUCUUCCAAUUGGCCAUGAUCAACUUCAUUCAUUUACUAGGGCAUUGCAGG<br>AAUAGUACCCUGAUGUGAGCUCUUCCCAGGGGGCGCCUAUCGCCG
N5.8U	UUUUUUUUGUGAUUCUUCCAAUUGGCCAUGAUCAACUUCAUUCAUUUACUAGGGCAUUGCAGGAAUA<br>GUACCCUGAUGUGAGCUCUUCCCAGGGGGCGCCUAUCGCCGUUCUUAUGGUUAGACGUAGGACCUUGC<br>UAACUUCUCUCACACAUUCUCUAUUUUGCAAUCUCUUUCAACUAAUUGAUACAGGGUCUGCCACAACC<br>UUCUCUAUCUGUUAUGACAGCAAGACAUCCAUUCUGAUAGAGAGUGUCCUAUCCCGACUUUCUCGCGA<br>GGGGUUACCACCGAGCGCCGACAUAGGAUUCAUUCUCUUUACACAUUAGAGUCAUCUUCUAACCCAUC<br>UGGCACUAC
N5.4U	UUUUGUGAUUCUUCCAAUUGGCCAUGAUCAACUUCAUUCAUUUACUAGGGCAUUGCAGGAAUAGUAC<br>CCUGAUGUGAGCUCUUCCCAGGGGGCGCCUAUCGCCGUUCUUAUGGUUAGACGUAGGACCUUGCUAAC<br>UUCUCUCACACAUUCUCUAUUUUGCAAUCUCUUUCAACUAAUUGAUACAGGGUCUGCCACAACCUUCU<br>CUAUCUGUUAUGACAGCAAGACAUCCAUUCUGAUAGAGAGUGUCCUAUCCCGACUUUCUCGCGAGGG<br>GUUACCACCGAGCGCCGACAUAGGAUUCAUUCUCUUUACACAUUAGAGUCAUCUUCUAACCCAUCUGG<br>CACUAC
